# Multi-Modal Fake News Detection via Bridging the Gap between Modals

**DOI:** 10.3390/e25040614

**Published:** 2023-04-04

**Authors:** Peng Liu, Wenhua Qian, Dan Xu, Bingling Ren, Jinde Cao

**Affiliations:** 1 School of Information Science and Engineering Yunnan University, Kunming 650500, China; 2 School of Mathematics, Southeast University, Nanjing 210096, China; 3 Yonsei Frontier Lab., Yonsei University, Seoul 03722, Republic of Korea

**Keywords:** multi-modal, fake news detection, caption-based, transformer

## Abstract

Multi-modal fake news detection aims to identify fake information through text and corresponding images. The current methods purely combine images and text scenarios by a vanilla attention module but there exists a semantic gap between different scenarios. To address this issue, we introduce an image caption-based method to enhance the model’s ability to capture semantic information from images. Formally, we integrate image description information into the text to bridge the semantic gap between text and images. Moreover, to optimize image utilization and enhance the semantic interaction between images and text, we combine global and object features from the images for the final representation. Finally, we leverage a transformer to fuse the above multi-modal content. We carried out extensive experiments on two publicly available datasets, and the results show that our proposed method significantly improves performance compared to other existing methods.

## 1. Introduction

The swift and widespread adoption of social media platforms such as Twitter, Weibo, and Facebook has made it increasingly difficult for the general populace to differentiate between authentic and fabricated news [1]. Fake news or misinformation [2] is a serious issue that has garnered significant attention in recent years due to its potential to spread quickly and cause harm through social media and other online platforms [3,4]. As a result, the public is increasingly concerned about the credibility of news, leading to an increase in the number of people seeking methods for detecting fake news [5].

Artificial intelligence (AI) is a rapidly developing field that has been applied in various fields, such as medicine, security, and the military. In recent years, numerous studies have been conducted to explore the use of AI for the detection of fake news [6,7,8,9]. Most methods for detecting fake news are based on text analysis [10,11,12,13]. However, narration, discussion, and evaluation of news content, as well as photographs and video clips, are also considered essential elements in distinguishing fake news. Therefore, methods that consider multiple types of data, known as multi-modal methods, tend to perform better than those that only consider a single type of data [14,15].

**Figure 1:**
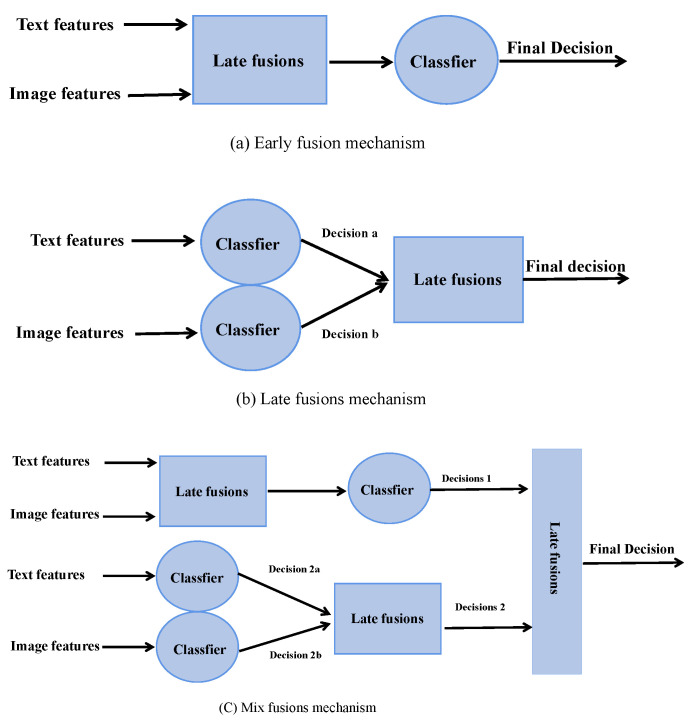
The previous methods can be distilled into the schema illustrated in the above figures [18,19,20].

The existing multi-modal methods can be distilled into the schema illustrated in Figure 1a–c. These methods used different fusion strategies to detect fake news. In addition, researchers also claimed that when the content of a news image is inconsistent with the text content, it indicates that the news is fake [16]. Based on this assumption, researchers encoded the image and text information of the news and calculated their similarity. However, after utilizing the CLIP [17] to analyze the similarity between the image and text quantitatively, we found no significant correlation between the similarity and the authenticity of the news (See Section 4.6). This may be because people prefer to use abstract images to express their views, but it does not mean that the news is fake. However, in the case of multi-modal fake news detection, the relationship between the image and text is highly complex. The conventional approaches may not capture the semantic interaction between image and text well.

**Figure 2:**
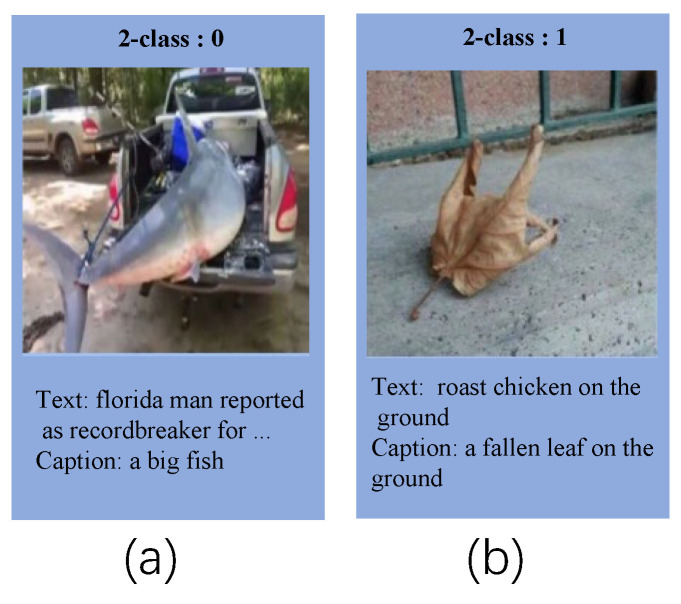
For (**a**), the text only mentions record breakers, not fish. Adding image description information, the text becomes “a big fish, florida man reported as record breaker for …”. This will bridge the gaps between the text and image. For (**b**), adding image description information will form conflicting statements with the original text, that is, “a fallen leaf on the ground, roast chicken on the ground.” The conflicting statements will help the model judge fake news.

**Figure 3:**
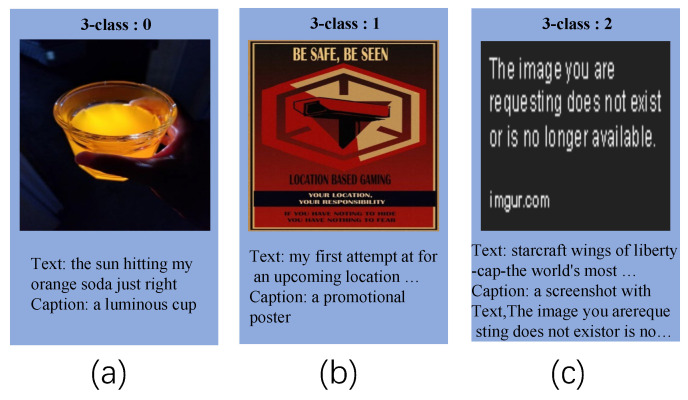
For (**a**), through caption information, we can conclude that the content of the text and image match. For (**b**), through caption information, we can conclude that the content of the text and image do not match. For (**c**), through caption information, we can conclude that the image originated from a screenshot, which is a false image.

**Figure 4:**
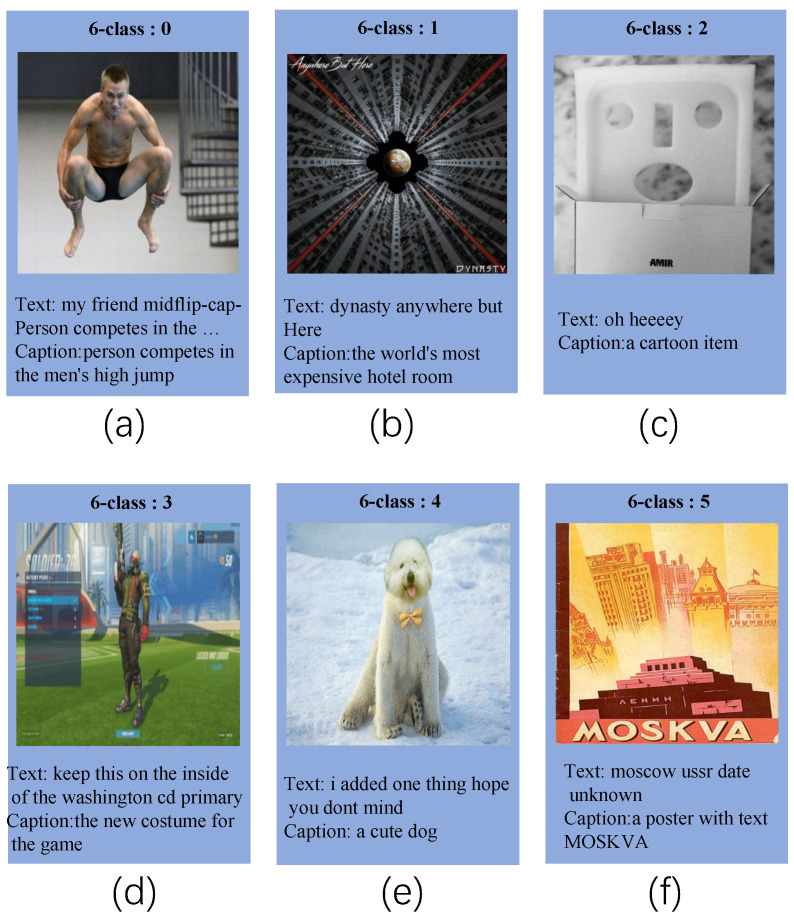
For (**a**), adding caption information will bridge the gaps between the text and image. For (**b**), The irony can be seen through the caption and the content in the text. For (**c**), through caption, our model can be inferred that the corresponding news is a story about fools. For (**d**), through caption, our model can be inferred that the text is generated by bot. For (**e**), through caption, our model can be inferred that the image in this news do not accurately support their text descriptions. For (**f**), through caption, our model can be inferred that the content has been purposely manipulated through manual editing or other forms. of alteration.

In this paper, we apply image description information to fake news detection. In the context of multi-modal fake news detection, we consider the follow cases, as shown in Figure 2, Figure 3 and Figure 4. Thus, we attempt to leverage image caption technology to generate image information and integrate it into the original text to bridge the gaps. In addition, the image holds crucial information that cannot be extracted through convolutional neural networks. As a result, we employ image caption technology to extract this vital data from the image and incorporate it as a supplement to the original text. On the whole, incorporating image description information can enhance semantic interaction between the text and image, which allows the model to extract multi-modal features more effectively, thereby improving the performance to distinguish fake news.

In addition, the previous methods only extracted the global features from the entire image through ResNet [21]. To enhance the exploration of the semantic relationship between text and image and optimize image utilization, we employ Faster R-CNN [22] to extract entity features and combine them with the global features to form comprehensive features.

**Figure 5:**
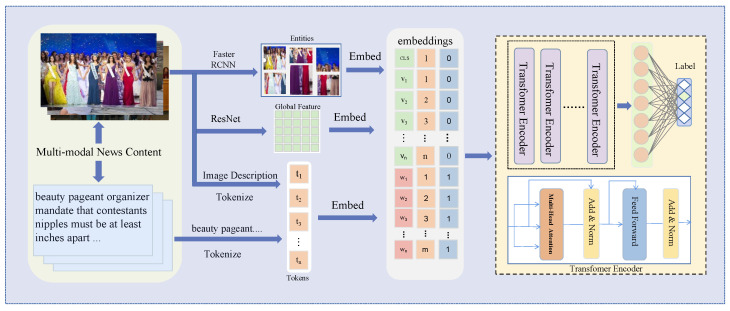
We utilize the ResNet and Faster R-CNN to extract global and entity features from images and BERT’s tokenizer to encode the text and image caption, which are then concatenated to form the final embedding. This embedding is subsequently fed into a multi-modal transformer for classification.

Overall, as shown in Figure 5, our approach addresses the limitations of the previous methods by considering the semantic interaction between image and text, improving the fusion of image and text information in the model.

The contributions in our paper can be succinctly summarized as follows:

- Our proposed method leverages a transformer architecture to effectively fuse the multi-modal data, thereby modeling the semantic relationships between images and texts.
- To capture the complex relationship between the image and text in multi-modal news, we analyze and propose utilizing image description information as a solution to enhance semantic interaction between the text and image.
- To further improve the exploration of the relationship between text and image and optimize image utilization, we combine entity features with global features to create comprehensive features.

The next of this paper is organized as follows: Section 2 reviews the related works about multi-modal fake news detection approach, and Section 3 presents the implementation of our proposed method in detail. Section 4 reports the results of our method. Finally, conclusions are presented in Section 5.

## 2. Related Works

This section briefly reviews previous related studies, emphasizing multi-modal fake news detection, image caption, and the application of the transformer to harness the multi-modal content.

### 2.1. Fake News Detection

News consumption is an important part of social life. The rapid development of information technology has made it possible for us to obtain more and more information. However, the rapid growth of information technology has also become a source of various problems. In particular, the rapid spread of fake news has become a severe problem [23,24,25]. Several multi-modal fake news detection methods have been proposed in the literature [15,26,27]. The MVAE [28] sought to learn the shared expression of text and visual modality through joint training of a VAE and classifiers for authentic and fake news. SpotFake [29] introduced the usage of BERT [30] in this framework. However, these methods present limitations in their ability to effectively model multi-modal interactions. In contrast, SAFE [31] calculated multi-modal inconsistency by comparing the similarity of modals generated through the creation of an image description and comparing it to the original text. The MCNN [32], on the other hand, mapped text and visual features to a shared space and calculated similarity through network weight sharing. Some researchers have also sought to address fake news detection through modal alignments, such as the attRNN [33], which used a neural network with an attention mechanism for image-text fusion. The MKEMN [34] sought to enhance semantic understanding through external knowledge. These methods have greatly promoted the advancement of fake news detection. However, the above methods do not account for potential discrepancies between the text and image.

Compared with the previous studies, we pay more attention to solving the problem of semantic mismatch between the image and text in fake news detection.

### 2.2. Image Caption

To demonstrate the potential of deep neural networks for image captioning, AICG [35] first introduced an encoder–decoder structure for this task. In recent years, there has been a growing interest in image captioning, with a number of works exploring new approaches to the problem. One trend that has emerged in the field is the use of attention mechanisms [36,37,38,39,40], which explored attention to effectively incorporate both global and local visual features in image captioning. Another trend in the field of image captioning focused on fine-grained details and object descriptions [41,42,43]. In recent studies, transformer models have also proven to be effective in several recent studies. Some methods use the transformer to effectively integrate visual and textual information [44,45,46,47,48]. In addition, studies have explored the use of a cross-modal transformer in image captioning [49,50], which integrated visual and textual information flexibly and effectively. These works demonstrate the progress made in the field of image captioning and highlight the importance of incorporating attention mechanisms and additional information into the models to improve performance.

In this paper, we utilized image caption technology to generate descriptive information for images and add them to the corresponding text, bridging the semantic gap between the images and text in multi-modal news.

### 2.3. Multi-Modal Transformers

A transformer [51] is an architecture based on attention mechanisms, which was first proposed in the field of natural language processing (NLP). In the field of NLP, BERT [30] conducted pre-training on the unlabeled text and achieved state-of-the-art performance in multiple NLP tasks by fine-tuning the output layer. Inspired by BERT, GPT-3 [52] pre-trained a super large-scale transformer model with 175 billion parameters. Without fine-tuning, GPT-3 model showed strong ability in various downstream tasks. The studies based on transformer has greatly promoted the development of the NLP field. The successful application of the transformer in the field of NLP attracted researchers and scholars to explore and try its application in other field. In recent years, multi-modal pre-training transformers have gained significant attention as they show promising results in various computer vision and NLP tasks. For example, ViLBERT [53], a joint model for vision-and-language tasks that were trained on large visual features and text captions. LXMERT [54] is another pre-trained model that leveraged both visual and textual features to perform tasks such as image captioning and visual question answering. VisualBERT [55] is a visual-linguistic pre-training model that uses both visual and linguistic features to perform tasks such as image captioning and visual question answering. These works highlight the effectiveness of multi-modal pre-training in improving the performance of models on a variety of tasks and domains and demonstrate the potential of these models in advancing the field of computer vision and natural language processing.

In this paper, we use a multi-model transformer framework to capture semantic information of images and text so as to improve the performance of fake news detection.

## 3. Method

### 3.1. Problem Definition

Multi-modal fake news detection aims to classify news items into their corresponding categories. Given a dataset $M=\left\{x_{1},x_{2},\ldots ,x_{n}\right\}$, where *n* represents the total size of the dataset, $x_{i}=\left\{t,i,y\right\}$ denotes a single news, where *t* denotes the text and *i* denotes the image, *y* belongs to $\left\{0,1,\ldots ,c\right\}$, representing the news ground-truth. We train our model to map the news to its corresponding ground-truth.

### 3.2. Model Overview

In this section, we present a comprehensive description of our proposed model, as shown in Figure 3, which can be divided into three parts. The first part, the embedding, projects the multi-modal data into a high-dimensional space where the following model components can effectively process it. The second part, the transformer, is a neural network that allows for efficient computation of self-attention mechanisms, thus enabling the model to capture cross-modal information in the multi-modal data effectively. In the third part, we mainly introduce classification loss. Together, these three parts form the foundation of our proposed method.

**Text embeddings** convert text to vectors. We first use the image caption technology [56] to generate the image description and insert it into the text corresponding to the image to form the final text. We then convert the text *T* into one-hot tokens to create these embeddings $T=\left[t_{1},t_{2},\ldots ,t_{m}\right]$, where *m* is the maximum length of the text sequence, and *t* is a one-hot vector. For the Fakeddit dataset, sentences are broken down into word sequences using spaces, whereas for the Weibo dataset, characters are treated as unit. We initiate a matrix and employ it to transform one-hot tokens into dense tokens. This process can be expressed as follows:

(1) $$T\Leftrightarrow \left[w_{1},w_{2},\ldots ,w_{m}\right]=\left[t_{1}W^{T},t_{2}W^{T},\ldots ,t_{m}W^{T}\right]$$

where $W^{T}\in R^{V\times H}$ is the text embedding matrix with the dimension of V×H, *V* represents the vocabulary length, and *H* represents the dimension of a dense token. In this part, we initialize the embedding using the pre-trained BERT model.

**Image embeddings** includes global feature and entity feature embeddings. For the global feature, we utilize ResNet to generate image features and convert them into sequences through a pooling strategy. We define the process as follows:

(2) $$\begin{array}{cc} F_{I} & =\mathrm{ResNet}(I)\\ {\bar{F}}_{I} & =\mathrm{AvgPool}\left(F_{I},g\right)\\ S_{V} & ={\bar{F}}_{I}W^{I}\\ S_{V} & \Leftrightarrow \left[v_{1},v_{2},\ldots ,v_{g}\right] \end{array}$$

where $F_{I}\in R^{7\times 7\times 2048}$ represents the global feature of the images extracted by ResNet. Then we apply average pooling to $F_{I}$ to create a sequence ${\bar{F}}_{I}\in R^{g\times 2048}$, where *g* defines the length of the sequence selected by our experience. After that, we use a matrix $W^{I}\in R^{2048\times H}$ to map the sequence to have the same dimension as the text embeddings. Finally, we denote $S_{V}\Leftrightarrow \left[v_{1},v_{2},\ldots ,v_{g}\right]$ as global image embeddings.

For the entity feature, a pre-trained Faster RCNN is used to generate entities $\left[R_{1},R_{2},\ldots ,R_{e}\right]$, where *e* is a hyper-parameters to control the number of entities in an image. Similar to global image embedding, we also use ResNet to extract the representation of each entity. We define this process as follows:

(3) $$\begin{array}{cc} \; & \left[{\bar{R}}_{1},{\bar{R}}_{2},\ldots ,{\bar{R}}_{e}\right]=\mathrm{ResNet}\left(\left[R_{1},R_{2},\ldots ,R_{e}\right]\right)\\ \; & \left[r_{1},r_{2},\ldots ,r_{e}\right]=\left[{\bar{R}}_{1}W^{E},{\bar{R}}_{2}W^{E},\ldots ,{\bar{R}}_{e}W^{E}\right] \end{array}$$

where $\bar{R_{i}}\in R^{2048}$ represents the entity features generated by ResNet. $W^{E}\in R^{2048\times H}$ represents a projection matrix, which maps the dimension of the entity feature to the same dimension as the global feature.

**Final embeddings** combine the embeddings mentioned above and incorporate position and type embeddings. Similar to BERT, we add two additional embeddings. We define this process as follows:

(4) $$\begin{array}{cc} \; & T=\left[w_{1}+w^{\mathrm{type}\;},w_{2}+w^{\mathrm{type}\;},\ldots ,w_{n}+w^{type}\right]+W^{\mathrm{pos}\;}\\ \; & V=\left[v_{1}+v^{\mathrm{type}\;},\ldots ,v_{g}+v^{\mathrm{type}\;},r_{1}+v^{\mathrm{type}\;},\ldots ,r_{e}+v^{\mathrm{type}\;}\right]+V^{\mathrm{pos}\;} \end{array}$$

(5) D=T⊕V

where $w^{\mathrm{type}\;}\in R^{H}$ represents the type of text embedding, $v^{\mathrm{type}}\in R^{H}$ represents the type of image embedding, $W^{\mathrm{pos}}\in R^{n\times H}$ represents the text position embedding, and $V^{\mathrm{pos}}\in R^{g\times H}$ represents the image position embedding, respectively. Finally, as described in formula 5, we concatenate *T* and *V* to form the multi-modal embedding *D*. We set the dimension of D to 768.

**A multi-modal transformer** is used to fuse the final embeddings *D* to obtain the representation. The transformer model is capable of representing sequence by computing correlations of elements in the sequence through the multi-head self-attention mechanism. Thus, we use the transformer in our method to fuse the cross-modal relationship between language and visual information, thus allowing for efficient interaction between cross-modal information, which can be defined as follows:

(6) $$\mathrm{Attention}\left(Q,K,V\right)=\mathrm{Softmax}\left(\frac{QK^{T}}{\sqrt{d_{k}}}\right)V$$

(7) $$\begin{array}{cc} \mathrm{MultiHead}(D) & =\mathrm{Concat}\left(h_{1},\ldots ,h_{n}\right)W^{O}\\ h_{i} & =\mathrm{Attention}\left(DW_{i}^{Q},DW_{i}^{K},DW_{i}^{V}\right) \end{array}$$

where Q, K, and V represent Query, Key, and Value, respectively, and $d_{k}$ represents the dimension of *K*. The $W^{Q}$, $W^{K}$, and $W^{V}$ are head projection matrixes. $W^{O}$ is used to aggregate the concatenated head. In our method, similar to BERT, we also use the first token in the transformer’s top layer to represent text and the image. Concat means directly concatenate all attention. Softmax represents a normalized exponential function.

**Classification Loss**: Given a mini-batch training data that contains *m* samples, $M=\{x_{1},x_{2},\ldots ,x_{m}\}$, for each data $x_{i}$ at the training step, we feed $x_{i}$ to go through the forward pass of the multi-modal transformer. Then, we can obtain the cls token output $CLS=\{cls_{1},cls_{2},\ldots ,cls_{m}\}$. Finally, we classify each $cls_{i}$, which can be expressed by:

(8) $$\begin{array}{c} P\left(y_{i}|cls_{i}\right)=\mathrm{Softmax}\left(cls_{i}W_{c}\right) \end{array}$$

where $W_{C}\in R^{H\times c}$ represents a projection matrix and *c* denotes the number of all categories. Softmax represents a normalized exponential function. For the fake news detection task, our goal is to train the model to minimize the negative log-likelihood loss, which the following equation can express:

(9) $$L_{nll}=\frac{1}{m}\sum_{i=1}^{m}-\log P\left(y_{i}|cls_{i}\right)$$

## 4. Experiments

### 4.1. Datasets

To demonstrate the performance of our method, we conducted evaluations using Fakeddit [57] and Weibo [33] datasets. The datasets are described below.

- **Fakeddit** [57] The Fakeddit dataset is a comprehensive dataset for fake news detection that has been collected from Reddit, a popular social media platform. The dataset comprises over one million samples and provides multi-grained labels covering text, images, metadata, and comments. It includes labeling information for 2, 3, and 6 categories, which offers increasing granularity of classification. For 2 categories, it determines whether a piece of news is real or fake. The others label information provides even greater specificity, enabling a more detailed classification of the news samples. All the samples are labeled through distant supervision and are further reviewed through multiple stages to ensure accuracy.
- **Weibo** [33] The Weibo dataset originates from China’s popular social media platform Weibo. The dataset contains both real and fake news samples. Each news item in the dataset includes the corresponding text, image, and label information.

The experimental evaluation of the proposed approach aligns with the methodology established in previous work [15]. The statistical information of the datasets is presented in Table 1.

### 4.2. Baseline Methods

We compared other methods, which were classified into two primary categories: single-modal and multi-modal approaches. The former only employs a single modal, such as text or image, for classification, whereas the latter uses both text and images for classification.

(1) Single-modal approaches

**Naive Bayes** exhibits a broad spectrum of utilization in various domains. This method can be applied to various text-based tasks, but in this study, it is specifically used to determine the category of a given piece of news text.

**BERT** [30] is a widely used natural language processing model that has achieved the best performance in a variety of downstream tasks.

**ResNet** [21] is a CNN architecture. It is widely utilized as a feature extractor in various tasks, particularly in the field of image classification.

(2) Multi-modal approaches

**EANN** [27] is a multi-modal approach used for news classification. It utilizes two different models to extract features, one for text and another for images. It uses a text-CNN to extract text features and VGG-19 to extract image features, then concatenates them and uses them to classify the news. Additionally, for a fair comparison, we used the same settings in [15] for the experiment.

**MVAE** [14] is a method used for feature extraction and representation learning in multi-modal tasks. It uses two different models, a bi-LSTM to extract text features and a VGG to extract image features. It then utilizes the VAE to attain the latent features. Furthermore, to mine the potential performance of the method, the VGG was replaced with the ResNet.

**BERT and ResNet** can still be considered as a strong baseline method. As a result of their widespread recognition, BERT and ResNet have been widely adopted in the fields of natural language processing and computer vision.

**MMBT** [58] used ResNet to extract image features and then convert them into image feature sequences. These image sequences are combined with text. After that, a transformer is applied to these sequences to encode and classify them.

**MTTV** [15] is a multi-modal method that uses two types of image features to model the news content so as to improve performance.

### 4.3. Evaluation Metrics

To validate our method, several evaluation metrics are used. The accuracy (Acc), precision (P), recall (R), and F1 score are used for binary classification tasks to evaluate the performance. For multiple classification tasks, accuracy is used as the evaluation metric. For all evaluation indicators, the higher the value, the better the performance of the model.

- Accuracy:
  (10) $$\;\mathrm{Acc}\;=\frac{TP+TN}{TP+TN+FP+FN}$$
- Precision:
  (11) $$\;P\;=\frac{TP}{TP+FP}$$
- Recall:
  (12) $$R=\frac{TP}{TP+FN}$$
- F1:
  (13) $$F_{1}=\frac{2\times TP}{2\times TP+FN+FP}$$

where TP refers to the number of cases in the actual sample that has been correctly identified as positive by the prediction, FP represents the number of negative samples that have been incorrectly identified as positive by the prediction. FN indicates the number of positive samples that have been incorrectly identified as negative by the prediction. TN represents the number of cases in the actual sample that has been correctly identified as negative by the prediction.

### 4.4. Implementation Details

Our proposed method is implemented using the Pytorch framework and executed on an NVIDIA 3090 graphics card. In our experiments, we used the pre-trained ResNet-152 model [21] as an image feature extractor. The transformer architecture employed in this study was based on the BERT structure as described in [30]. A batch size of 32 is used for both the Weibo and Fakeddit datasets. The Adam [59] optimizer with a learning rate of 5 × 10${}^{-5}$ is utilized to optimize the model. The number of global features *g* is set to 5. The number of entity features *e* is set to 10. In addition, we also extract text from the image to supplement image description information in Weibo datasets, as a large proportion of images in the datasets contain text.

### 4.5. Comparison Experiments

As shown in Table 2 and Table 3, the experimental results demonstrated that the multi-modal methods outperform the single-mode methods. It is possible that the complexity of interdependent relationships between the image and text information within news items contributes to the improvement in classification performance. The utilization of both text and image information may provide a more comprehensive understanding of the content. In other words, this can be attributed to the fact that the multi-modal information provides different viewpoints which complement each other, resulting in an improvement in performance.

We can also observe that our method outperforms other multi-modal methods, which may be due to the ability of our model to extract the features that are beneficial for fake news detection by bridging the gaps between the text and visual information. In addition, the Fakeddit dataset provides 2-class, 3-class, and 6-class labels for the news samples, and our method performed well on all levels of granularity. This indicated that our method is able to generalize well to different levels of complexity and different types of fake news. In other words, our proposed method for detecting fake news by integrating more features is effective and outperforms existing others. This is because our method is able to learn a representation that effectively discriminates between real and fake news by adding image description and entity-level features.

### 4.6. Relationship of Cross-Modal

**Figure 6:**
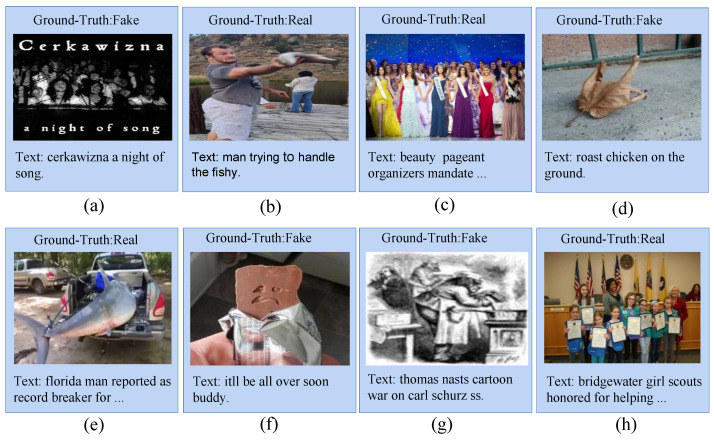
For the examples of multi-modal news entities, we randomly sampled 4 real news and 4 fake news from the Fakedit dataset, respectively. For each instance, the top of the image is its label information, and the bottom is the text information. (**b**,**c**,**e**,**h**) represent real news. (**a**,**d**,**f**,**g**) represent fake news.

In this section, we aimed to investigate the relationship between cross-modal similarity and news authenticity. As shown in Figure 6, we randomly sampled 4 real news and 4 fake news from the datasets, respectively. We used the CLIP pre-training model to calculate the cross-modal similarity of the sampled news and normalized the similarity between them. To eliminate the impact of different image encoders in CLIP on the results, we used two image encoders: RN50 and ViT-B/32.

**Figure 7:**
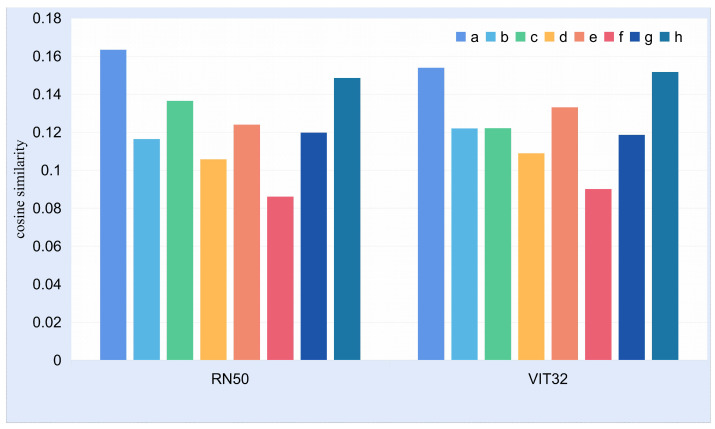
This figure shows the cross-modal similarity between the text and the image in Figure 6. RN50 means that the image encoding used in CLIP is RN50, and VIT32 means that ViT-B/32 is used as the encoder.

The results are shown in Figure 7. Through analysis, we found that the cross-modal similarity of fake news may also be higher than that of real news. In other words, there is no significant difference between fake news and real news in terms of cross-modal similarity. In the real world, people often express some abstract concepts, which often lack matching visual images, making it difficult to use cross-modal similarity to distinguish the authenticity of the news. In this case, we need to incorporate information from the image description to implement fake news detection.

### 4.7. The Impact of Image Caption

In this section, we analyzed why introducing image description information can improve the performance of the model. We analyzed the impact of the introduced image description information on the model prediction results. Specifically, we first obtained the output results of the model when introducing image description information. Then, we used the transformer’s mask mechanism to mask image description information and obtained the output results.

**Figure 8:**
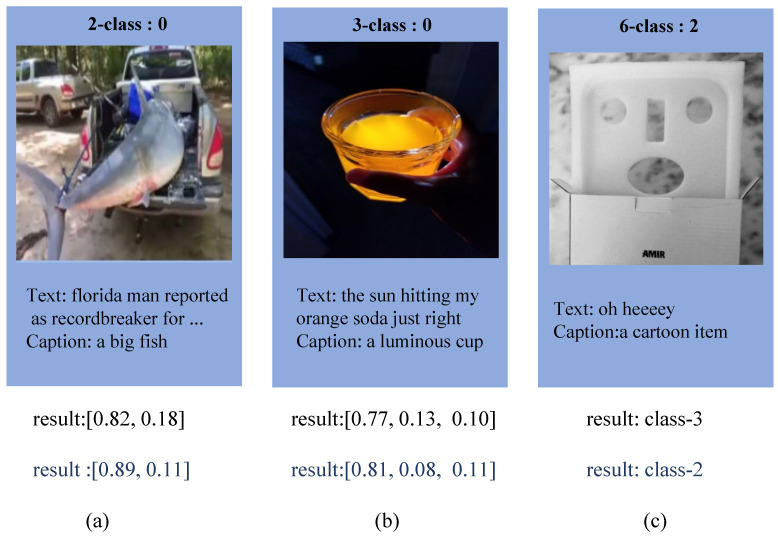
The impact of introducing the image caption on the prediction results of the model. The blue results indicate the prediction results after the introduction of the image caption. Introducing image caption information can enhance the confidence of the results for subgraphs (**a**,**b**), whereas for subgraph (**c**), the predicted result is rectified upon the inclusion of image caption information.

As shown in Figure 8, we find that under the condition of masking the image caption, the prediction results of the model will appear in two situations: (1) the prediction confidence of the model decreases. (2) The prediction results of the model may have errors. In the first case, the introduction of image description information may serve as a supplementary information to the text, thereby improving the predictive confidence of the model. In the second case, introducing image description information may bridge the semantic gap between image and text, thereby improving the prediction accuracy of the model.

### 4.8. The Impact of Local Regions

In this section, we first prove that it is impossible to extract fine-grained image local regions information from the global information of the image. We designed the following experiment: First, we used a transformer to extract the global feature of a image, while using the extracted features for region classification. We found that the performance of region classification using global features of images is poor. Therefore, using global features alone cannot achieve fine-grained alignment between image regions and text.

**Figure 9:**
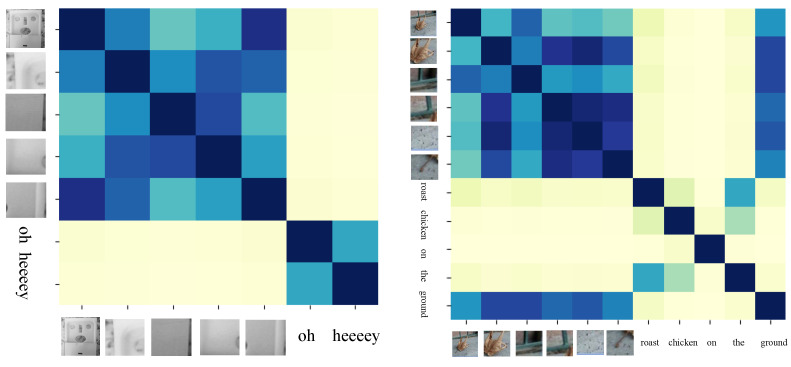
The text and image information in the left image are almost unrelated. However, in the image on the right, the word “ground” has a strong correlation with the small local regions corresponding to the image containing ground.

Then, we visualized the relationship between image regions and text by analyzing the weights of the transformer’s attention. The experimental results are shown in Figure 9. We found that the corresponding weights of the image regions described in the text is large, whereas the weights of the image regions not mentioned in the text is small. Therefore, although small local regions may have false connections with text, the corresponding attention weights for these regions are small. Therefore, these false connections will not hinder the performance of the model. In addition, we found that the weight information corresponding to some regions is equivalent to that of global information, indicating that providing local regions information and global information can complement each other, further improving the performance of the model.

In the ablation section of our experiment, removing global information can lead to a decrease in model performance, indicating that providing local regions information can indeed improve model performance.

### 4.9. Image Caption on Single-Modal

In this section, we investigated the impact of incorporating image description information into a single-modal for fake news detection. We presented two variants of the single-modal approach. The first variant used both ResNet and image description information as inputs to the transformer to examine the effect of adding image description information. The second variant incorporated the image description into the original text to assess whether this addition improved the performance.

The results of the experiments are shown in Table 4 and Table 5, which demonstrated that incorporating image description information can significantly improve the performance of fake news detection in the first variant. This revealed that utilizing only a convolutional neural network to extract image features may not fully exploit the information presented in the image. By integrating image description, the model can leverage more semantic information from the image to improve its performance. Additionally, in the second variant, incorporating image description into the original text can also improve the model performance. The potential cause for this could be attributed to the generated conflicting statements, which serve as useful information in detecting fake news, thus improving performance.

### 4.10. Ablation Experiments

In this part, we demonstrated how different components contribute to the model in the learning process. We compared the performance of our proposed method after specifically removing the global features (GF), entity features (EF), and image caption (IC), respectively. The comparison results are presented in Table 6 and Table 7. The results demonstrated that our proposed method achieved superior performance across all measurements on two datasets in most cases. This suggested that the utilization of entity features and image caption information are effective in providing representations that are conducive to classification. Additionally, the results also revealed that lacking image caption information resulted in a greater decrease in performance compared to the absence of entity features. This demonstrated that image caption information is crucial in enhancing the model’s performance.

## 5. Conclusions

In this paper, we propose bridging the semantic gap between text and images by utilizing image description information generated from image caption technology. Furthermore, we optimize the representation of images by combining entity features with global features. To better capture multi-modal semantic information, we leverage a transformer to fuse the above contents. The extensive experimentation shows that the proposed method significantly improves performance when compared to other existing methods. In future work, we aim to extract more information from images and text to bridge the semantic gaps.

## 6. Implications for Future Studies

The existing fake news detection methods often focus only on supervised methods with sufficient annotated data. However, in the real world, a large amount of data is often not annotated due to high annotation costs or time constraints, leading to insufficient or no annotated data. Therefore, supervised methods are often not suitable for real-world applications. As a result, unsupervised or weakly supervised methods are needed in real-world scenarios. In future work, we plan to extend our methods to the field of unsupervised learning.

## Figures and Tables

**Table 1:** Details of Fakeddit and Weibo datasets. R and F mean real and fake, respectively.

Dataset	Train (No.)	Validate (No.)	Test (No.)	Label	R:F
Fakeddit	563,523	59,283	59,257	2/3/6	2:3
Weibo	7183	-	1840	2	1:1

**Table 2:** Comparative results on the Fakeddit. Bold text in the table indicates the best results.

Method		2-Class				3-Class	6-Class
		Acc	P	R	F1	Acc	Acc
Single-modal	Naive Bayes	0.7933	0.8139	0.8522	0.8326	0.7750	0.6469
	BERT	0.8788	0.9147	0.8814	0.8977	0.8731	0.8070
	ResNet	0.7534	0.8032	0.7832	0.7911	0.7442	0.6936
Multi-modal	EANN	0.8750	0.9043	0.8811	0.8926	0.8702	0.8319
	MVAE	0.8875	0.9011	0.9139	0.9074	0.8838	0.8413
	BERT and ResNet	0.8942	0.9124	0.9122	0.9123	0.8926	0.8502
	MMBT	0.9111	0.9274	0.9251	0.9263	0.9058	0.8830
	MTTV	0.9188	0.9348	0.9303	0.9325	0.9162	0.8982
	Our	**0.9251**	**0.9383**	**0.9374**	**0.9379**	**0.9221**	**0.9057**

**Table 3:** Comparative results on the Weibo. Bold text in the table indicates the best results.

Method		Acc	P	R	F1
Single-modal	Naive Bayes	0.7130	0.6685	0.8213	0.7371
	BERT	0.8538	0.8427	0.8624	0.8524
	ResNet	0.6451	0.6483	0.6016	0.6241
Multi-modal	EANN	0.7950	0.8060	0.7950	0.8000
	MVAE	0.8240	0.8540	0.7690	0.8090
	BERT and ResNet	0.8603	0.9055	0.7980	0.8484
	MMBT	0.8658	0.8733	0.8491	0.8610
	MTTV	0.8766	0.8616	0.8912	0.8762
	Our	**0.8886**	**0.8692**	**0.9201**	**0.8939**

**Table 4:** The effectiveness of image caption on a single-modal on Fakeddit datasets. IC means image caption information. Bold text in the table indicates the best results.

Modal	2-Class				3-Class	6-Class
	Acc	P	R	F1	Acc	Acc
Text	0.8788	**0.9147**	0.8814	0.8977	0.8731	0.8070
Text w/ IC	**0.8940**	0.9141	**0.9099**	**0.9120**	**0.8881**	**0.8134**
Image	0.7534	0.8032	0.7832	0.7911	0.7442	0.6936
Image w/ IC	**0.8103**	**0.8626**	**0.8154**	**0.8384**	**0.8016**	**0.7600**

**Table 5:** The effectiveness of image caption on a single-modal on Weibo datasets. IC means image caption information. Bold text in the table indicates the best results.

Modal	Acc	P	R	F1
Text	0.8538	0.8427	**0.8624**	0.8524
Text w/ IC	**0.8571**	**0.8603**	0.8594	**0.8598**
Image	0.6451	0.6483	0.6016	0.6241
Image w/ IC	**0.6565**	**0.6574**	**0.6826**	**0.6698**

**Table 6:** Ablation results on Weibo. GF, EF and IC represent global features, entity features, and image caption information, respectively. Bold text in the table indicates the best results.

Configuration	Acc	P	R	F1
Remove GF	0.8815	0.8523	0.9286	0.8896
Remove EF	0.8820	0.8525	0.9297	0.8894
Remove IC	0.8766	0.8429	**0.9318**	0.8889
Our	**0.8886**	**0.8692**	0.9201	**0.8939**

**Table 7:** Ablation results on Fakeddit. GF, EF and IC represent global features, entity features, and image caption information, respectively. Bold text in the table indicates the best results.

Configuration	2-Class				3-Class	6-Class
	Acc	P	R	F1	Acc	Acc
Remove GF	0.9205	0.9221	**0.9482**	0.9350	0.9202	0.9018
Remove EF	0.9211	0.9305	0.9394	0.9349	0.9206	0.9031
Remove IC	0.9139	0.9212	0.9374	0.9293	0.9169	0.8913
Our	**0.9251**	**0.9383**	0.9374	**0.9379**	**0.9221**	**0.9057**

## Data Availability

Not applicable.

## References

[1] Shu K., Sliva A., Wang S., Tang J., Liu H. (2017). Fake news detection on social media: A data mining perspective. ACM SIGKDD Explor. Newsl..

[2] Scheufele D.A., Krause N.M. (2019). Science audiences, misinformation, and fake news. Proc. Natl. Acad. Sci. USA.

[3] Allcott H., Gentzkow M. (2017). Social media and fake news in the 2016 election. J. Econ. Perspect..

[4] Rocha Y.M., de Moura G.A., Desidério G.A., de Oliveira C.H., Lourenço F.D., de Figueiredo Nicolete L.D. (2021). The impact of fake news on social media and its influence on health during the COVID-19 pandemic: A systematic review. J. Public Health.

[5] Vosoughi S., Roy D., Aral S. (2018). The spread of true and false news online. Science.

[6] Kaliyar R.K., Goswami A., Narang P. (2021). EchoFakeD: Improving fake news detection in social media with an efficient deep neural network. Neural Comput. Appl..

[7] Inan E. (2022). ZoKa: A fake news detection method using edge-weighted graph attention network with transfer models. Neural Comput. Appl..

[8] Nassif A.B., Elnagar A., Elgendy O., Afadar Y. (2022). Arabic fake news detection based on deep contextualized embedding models. Neural Comput. Appl..

[9] Singh B., Sharma D.K. (2022). Predicting image credibility in fake news over social media using multi-modal approach. Neural Comput. Appl..

[10] Liu Y., Wu Y.F. Early detection of fake news on social media through propagation path classification with recurrent and convolutional networks. Proceedings of the AAAI Conference on Artificial Intelligence.

[11] Zhou X., Zafarani R. (2019). Network-based fake news detection: A pattern-driven approach. ACM SIGKDD Explor. Newsl..

[12] Singhania S., Fernandez N., Rao S. (2017). 3han: A deep neural network for fake news detection. Proceedings of the Neural Information Processing: 24th International Conference, ICONIP 2017.

[13] Ma J., Gao W., Mitra P., Kwon S., Jansen B.J., Wong K.F., Cha M. (2016). Detecting Rumors from Microblogs with Recurrent Neural Networks.

[14] Khattar D., Goud J.S., Gupta M., Varma V. Mvae: Multimodal variational autoencoder for fake news detection. Proceedings of the World Wide Web Conference.

[15] Wang B., Feng Y., Xiong X.C., Wang Y.H., Qiang B.H. (2022). Multi-modal transformer using two-level visual features for fake news detection. Appl. Intell..

[16] Zhou X., Wu J., Zafarani R. (2020). Similarity-Aware Multi-modal Fake News Detection. Proceedings of the Advances in Knowledge Discovery and Data Mining: 24th Pacific-Asia Conference, PAKDD 2020, Singapore, 11–14 May 2020, Proceedings, Part II.

[17] Radford A., Kim J.W., Hallacy C., Ramesh A., Goh G., Agarwal S., Sastry G., Askell A., Mishkin P., Clark J. Learning transferable visual models from natural language supervision. Proceedings of the International Conference on Machine Learning, PMLR.

[18] Segura-Bedmar I., Alonso-Bartolome S. (2022). Multimodal fake news detection. Information.

[19] Abdali S. (2022). Multi-modal Misinformation Detection: Approaches, Challenges and Opportunities. arXiv.

[20] Alam F., Cresci S., Chakraborty T., Silvestri F., Dimitrov D., Martino G.D.S., Shaar S., Firooz H., Nakov P. (2021). A survey on multimodal disinformation detection. arXiv.

[21] He K., Zhang X., Ren S., Sun J. Deep residual learning for image recognition. Proceedings of the IEEE Conference on Computer Vision and Pattern Recognition.

[22] Ren S., He K., Girshick R., Sun J. (2015). Faster r-cnn: Towards real-time object detection with region proposal networks. Adv. Neural Inf. Process. Syst..

[23] Loos E., Nijenhuis J. (2020). Consuming Fake News: A Matter of Age? The perception of political fake news stories in Facebook ads. Proceedings of the Human Aspects of IT for the Aged Population. Technology and Society: 6th International Conference, ITAP 2020, Held as Part of the 22nd HCI International Conference, HCII 2020, Copenhagen, Denmark, 19–24 July 2020; Proceedings, Part III 22.

[24] Gao Q., Zhou J. (2020). Human Aspects of IT for the Aged Population. Technologies, Design and User Experience: 6th International Conference, ITAP 2020, Held as Part of the 22nd HCI International Conference, HCII 2020, Copenhagen, Denmark, 19–24 July 2020, Proceedings, Part I.

[25] Zhou J., Salvendy G. (2016). Human Aspects of IT for the Aged Population. Design for Aging: Second International Conference, ITAP 2016, Held as Part of HCI International 2016, Toronto, ON, Canada, 17–22 July 2016, Proceedings, Part I.

[26] Zhang T., Wang D., Chen H., Zeng Z., Guo W., Miao C., Cui L. BDANN: BERT-based domain adaptation neural network for multi-modal fake news detection. Proceedings of the 2020 international joint conference on neural networks (IJCNN).

[27] Wang Y., Ma F., Jin Z., Yuan Y., Xun G., Jha K., Su L., Gao J. Eann: Event adversarial neural networks for multi-modal fake news detection. Proceedings of the 24th ACM SIGKDD International Conference on Knowledge Discovery & Data Mining.

[28] Qi P., Cao J., Yang T., Guo J., Li J. Exploiting multi-domain visual information for fake news detection. Proceedings of the 2019 IEEE International Conference on Data Mining (ICDM).

[29] Singhal S., Shah R.R., Chakraborty T., Kumaraguru P., Satoh S. Spotfake: A multi-modal framework for fake news detection. Proceedings of the 2019 IEEE Fifth International Conference on Multimedia Big Data (BigMM).

[30] Devlin J., Chang M.W., Lee K., Toutanova K. (2018). Bert: Pre-training of deep bidirectional transformers for language understanding. arXiv.

[31] Zhou X., Wu J., Zafarani R. (2020). Safe: Similarity-aware multi-modal fake news detection (2020). Advances in Knowledge Discovery and Data Mining. PAKDD 2020.

[32] Li Q., Hu Q., Lu Y., Yang Y., Cheng J. (2020). Multi-level word features based on CNN for fake news detection in cultural communication. Pers. Ubiquitous Comput..

[33] Jin Z., Cao J., Guo H., Zhang Y., Luo J. Multimodal fusion with recurrent neural networks for rumor detection on microblogs. Proceedings of the 25th ACM International Conference on Multimedia.

[34] Zhang H., Fang Q., Qian S., Xu C. Multi-modal knowledge-aware event memory network for social media rumor detection. Proceedings of the 27th ACM international conference on multimedia.

[35] Vinyals O., Toshev A., Bengio S., Erhan D. Show and tell: A neural image caption generator. Proceedings of the IEEE Conference on Computer Vision and Pattern Recognition.

[36] Anderson P., He X., Buehler C., Teney D., Johnson M., Gould S., Zhang L. Bottom-up and top-down attention for image captioning and visual question answering. Proceedings of the IEEE Conference on Computer Vision and Pattern Recognition.

[37] Tran A., Mathews A., Xie L. Transform and tell: Entity-aware news image captioning. Proceedings of the IEEE/CVF Conference on Computer Vision and Pattern Recognition.

[38] Chen L., Zhang H., Xiao J., Nie L., Shao J., Liu W., Chua T.S. Sca-cnn: Spatial and channel-wise attention in convolutional networks for image captioning. Proceedings of the IEEE Conference on Computer Vision and Pattern Recognition.

[39] Lee K.H., Chen X., Hua G., Hu H., He X. Stacked cross attention for image-text matching. Proceedings of the European conference on computer vision (ECCV).

[40] Huang L., Wang W., Chen J., Wei X.Y. Attention on attention for image captioning. Proceedings of the IEEE/CVF International Conference on Computer Vision.

[41] Chowdhury P.N., Sain A., Bhunia A.K., Xiang T., Gryaditskaya Y., Song Y.Z. (2022). FS-COCO: Towards understanding of freehand sketches of common objects in context. Proceedings of the Computer Vision–ECCV 2022: 17th European Conference, Tel Aviv, Israel, 23–27 October 2022; Proceedings, Part VIII.

[42] Feng Q., Wu Y., Fan H., Yan C., Xu M., Yang Y. (2020). Cascaded revision network for novel object captioning. IEEE Trans. Circuits Syst. Video Technol..

[43] Wu J., Chen T., Wu H., Yang Z., Luo G., Lin L. (2020). Fine-grained image captioning with global-local discriminative objective. IEEE Trans. Multimed..

[44] Cornia M., Stefanini M., Baraldi L., Cucchiara R. Meshed-memory transformer for image captioning. Proceedings of the IEEE/CVF Conference on Computer Vision and Pattern Recognition.

[45] Liu J., Wang K., Xu C., Zhao Z., Xu R., Shen Y., Yang M. Interactive dual generative adversarial networks for image captioning. Proceedings of the AAAI Conference on Artificial Intelligence.

[46] Deng C., Ding N., Tan M., Wu Q. (2020). Length-controllable image captioning. Proceedings of the Computer Vision–ECCV 2020: 16th European Conference.

[47] Zhang Z., Wu Q., Wang Y., Chen F. (2021). Exploring region relationships implicitly: Image captioning with visual relationship attention. Image Vis. Comput..

[48] Liu B., Wang D., Yang X., Zhou Y., Yao R., Shao Z., Zhao J. Show, deconfound and tell: Image captioning with causal inference. Proceedings of the IEEE/CVF Conference on Computer Vision and Pattern Recognition.

[49] Wu S., Zha Z.J., Wang Z., Li H., Wu F. Densely Supervised Hierarchical Policy-Value Network for Image Paragraph Generation. Proceedings of the IJCAI.

[50] Zhao W., Wu X., Luo J. (2020). Cross-domain image captioning via cross-modal retrieval and model adaptation. IEEE Trans. Image Process..

[51] Vaswani A., Shazeer N., Parmar N., Uszkoreit J., Jones L., Gomez A.N., Kaiser Ł., Polosukhin I. (2017). Attention is all you need. Adv. Neural Inf. Process. Syst..

[52] Brown T., Mann B., Ryder N., Subbiah M., Kaplan J.D., Dhariwal P., Neelakantan A., Shyam P., Sastry G., Askell A. (2020). Language models are few-shot learners. Adv. Neural Inf. Process. Syst..

[53] Lu J., Batra D., Parikh D., Lee S. (2019). Vilbert: Pretraining task-agnostic visiolinguistic representations for vision-and-language tasks. Adv. Neural Inf. Process. Syst..

[54] Lu J., Goswami V., Rohrbach M., Parikh D., Lee S. 12-in-1: Multi-task vision and language representation learning. Proceedings of the IEEE/CVF Conference on Computer Vision and Pattern Recognition.

[55] Li L.H., Yatskar M., Yin D., Hsieh C.J., Chang K.W. (2019). Visualbert: A simple and performant baseline for vision and language. arXiv.

[56] Mokady R., Hertz A., Bermano A.H. (2021). Clipcap: Clip prefix for image captioning. arXiv.

[57] Nakamura K., Levy S., Wang W.Y. Fakeddit: A New Multimodal Benchmark Dataset for Fine-grained Fake News Detection. Proceedings of the Twelfth Language Resources and Evaluation Conference.

[58] Kiela D., Bhooshan S., Firooz H., Perez E., Testuggine D. (2019). Supervised multimodal bitransformers for classifying images and text. arXiv.

[59] Kingma D.P., Ba J. Adam: A method for stochastic optimization. Proceedings of the 3rd International Conference for Learning Representations.

